# Patient and Clinician Reported Outcomes of the Inframammary Incision “Short Scar Technique” in Primary Breast Augmentation

**DOI:** 10.1093/asjof/ojad003

**Published:** 2023-01-12

**Authors:** Paolo Montemurro, Mubashir Cheema, Tommaso Pellegatta, Per Hedén

**Affiliations:** private practice in Stockholm, Sweden; Department of Plastic Surgery, University Hospitals Birmingham, United Kingdom; private practice in Stockholm, Sweden; private practice in Stockholm, Sweden

## Abstract

**Background:**

Breast augmentation is a common aesthetic surgery procedure and surgeons are constantly trying to develop techniques that help improve patients' outcome. One of the most important aspects is achieving a favorable scar. The “traditional” breast augmentation scar is in the inframammary fold (IMF), whereas trans-axillary and trans-umbilical approaches have been described as an attempt to move the “location” of the scar and make it less noticeable. Nonetheless, relatively little attention has been paid to improving the IMF scar, which remains the most commonly used scar for silicone implants.

**Objectives:**

The authors have previously described a technique that uses an insertion sleeve and custom-made retractors to allow implant insertion through a shorter IMF scar. However, at the time, the authors did not evaluate the quality of the scar and patient satisfaction. In this manuscript, the authors describe patient and clinician-reported outcomes for this short scar technique.

**Methods:**

All consecutive female patients, undergoing primary aesthetic breast augmentation with symmetric implants were included in this review.

**Results:**

Three different scar-assessment scales demonstrated good results at 1-year postop, as well as the good correlation between patient-reported and clinician-observed scores. BREAST-Q subscale for overall satisfaction also demonstrated good overall patient satisfaction.

**Conclusions:**

Besides providing an added aesthetic value to the result of breast augmentation, a shorter scar may also appeal to patients who are concerned about the size and quality of postoperative scars and like to search for “before and after” pictures prior to scheduling consultations.

**Level of Evidence: 4:**

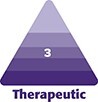

Breast augmentation is one of the most common aesthetic surgery operations.^1^ The Aesthetic Society's figures show that there were 364,753 breast augmentation procedures carried out in the United States in 2021^1^ and this was one of the 5 most common aesthetic surgery procedures in the United States. The considerable evidence base thus accumulated helps the surgeons focus on various important aspects of this operation in order to improve patient outcome. One of these aspects is certainly the final scar. In 1974, Eiseman^2^ described the trans-axillary approach while in 1993, Johnson and Christ^3^ popularized the trans-umbilical approach to breast augmentation, in an attempt to hide the operation scar.^4^ Nonetheless, there appears to be little literature devoted to improving upon the traditional inframammary fold (IMF) scar, even when it continues to be the most common access incision for silicone implants.

The final appearance of the scar after breast augmentation does indeed have clinical relevance now when more and more patients want to see representative “before and after” pictures of the operation in the current age of social media. A recent study showed that an increasing number of patients are looking for information on the internet and social media before booking a consultation with the plastic surgeon.^3^

With the aim of improving upon the traditional IMF scar for breast augmentation, we have previously described a short scar technique and its short-term results.^5^ In this manuscript, we provide longer term results with patient and physician-reported outcomes of the technique.

## METHODS

A total of 78 consecutive female patients who underwent primary breast augmentation between January 2019 and August 2019 by the first author, were reviewed. Preoperative planning, implant selection, surgical technique, and postoperative care followed the principles of the Akademikliniken (AK) method.^4–6^ The IMF incision was also positioned according to the AK method, ie, calculating that the scar would end up exactly at the level of the new IMF (the new nipple-IMF distance was found by adding to the implant's low ventral curvature [LVC] to half of the local tissue pinch thickness). All patients presented symmetrical breasts and received the same implant on each side through an IMF incision and a dual-plane dissection technique. Implants were inserted using an insertion sleeve and custom-made retractors.^5^ The IMF was reconstructed with barbed sutures according to the previously published “4-layer wound closure” technique.^6^ The Declaration of Helsinki guidelines were followed and written informed consent was obtained preoperatively.

Patients were reviewed at 1 week, 2 weeks, 3 months, 6 months, and 1 year. At 1 year postoperative, patients were asked to fill in the Patient and Observer Scar Assessment Scale (POSAS; patient scale)^7,8^ and the BREAST-Q^9,10^ (satisfaction with breasts subscale) questionnaires. The first author also performed scar assessment using the Vancouver Scar Scale (VSS), the POSAS (observer scale) and the Manchester Scar Scale (MSS). The resulting data were analyzed using the “R” statistical package.^11^ The distribution of each variable was assessed using the Shapiro–Wilk normality test and (in absence of normality) Kendall rank correlation coefficient was used for the rank-based measure of association between different scores.

## RESULTS

Seventy-eight female patients' clinical records were reviewed. Their mean age was 37.3 years (range, 24-62 years). The mean follow-up was 14.7 months (range, 12-65 months). There were 4 unilateral cases (5.1%) of minor superficial wound healing problems which were resolved within 2 weeks with conservative management.

The mean patient POSAS score for the right breast was 6.82 (median 6, range 6-20) and for the left breast was 6.51 (median 6, range 6-13; Table). The mean POSAS observer score for the right breast was 6.91 (median 6, range 6-14) and for the left breast was 6.56 (median 6, range 6-15). The mean VSS score for the right breast was 0.26 (median 0, range 0-3) and for the left breast was 0.10 (median 0, range 0-2). The MSS mean score for the right breast was 5.42 (median 5, range 5-8) and for the left breast was 5.23 (median 5, range 5-8; Table). The mean score with the “satisfaction with breasts” subscale of BREAST-Q was 83.86 (median 85, range 59-100).

**Table.** Data Table of the Different Assessment Scales Used

Statistic	Patient assessment			Observer assessment					
	1. Satisfaction with breasts	POSAS patient scale right (6-60)	POSAS patient scale left (6-60)	POSAS observer scale right (6-60)	POSAS observer scale left (6-60)	Vancouver scale right (0-13)	Vancouver scale left (0-13)	Manchester scale right (5-18)	Manchester scale left (5-18)
Median	85	6	6	6	6	0	0	5	5
Average	83.86	6.82	6.51	6.91	6.56	0.26	0.10	5.42	5.23
Min	59.0	6.0	6.0	6.0	6.0	0.0	0.0	5.0	5.0
Max	100	20.0	13.0	13.0	13.0	3.0	2.0	8.0	8.0

Higher score is better for the BREAST-Q subscale. For all the other subscales, lower score is better.

The variables were not in a normal distribution as determined by the Shapiro–Wilk test, therefore Kendall rank correlation test was used to measure of association. There was a significant correlation between the patient and observer components of the POSAS score (*P* < 0.001 for both right and left sides). There was also statistically significant correlation between patient-reported POSAS score, and the Vancouver Scar Assessment (*P* < 0.01) and Manchester Scar (*P* < 0.01) scales for either side.

## DISCUSSION

An estimated 100 million patients acquire postoperative scars in the developed world each year.^12^ Every tissue injury initiates the process of wound healing that is aimed at restoring tissue integrity. In breast augmentation surgery, periareolar,^13^ trans-axillary,^2^ or trans-umblical^3^ incisions aim to hide the operative scar where it is least noticeable. However, the inframammary incision continues to be used commonly, especially with silicone implants, as it allows for easier insertion and direct visualization of the pocket.^14–18^ Possibly due to the size constraints of the silicone implants, there has been little written in the literature about improving upon the length of this incision.

In our previous work,^5^ we described that with the use of an insertion funnel and custom-made narrow blade retractors, a silicone implant (mean 322.4 cc and range 165–495 cc, vs implants of mean volume 325.0 cc and range 215–450 cc with conventional technique) can be inserted through a short IMF incision (mean length 35.5 mm ± 2.1 mm vs 46.2 ± 3.2 mm with conventional technique). This did not affect short-term complications or risk of implant damage.^5^ However, the scar quality and patient satisfaction with overall outcomes were not evaluated. That left open to critique about possible impaired healing and poor scarring (from trauma during insertion) or poor aesthetics (from potential difficulties in performing an appropriate dissection through a smaller incision). In this review, we have aimed to address both of these concerns. The scar-assessment scales specifically looked at the quality and characteristics of the operative scars, while the BREAST-Q's “Satisfaction with breasts” subscale addressed the second question (whether operating through a smaller incision somehow affects the overall aesthetic outcome).

### Scar Assessment Scales

While there is no consensus on the best method to completely evaluate scar characteristics, an ideal scar-assessment tool should encompass the following features: non**-**invasive, accurate, reproducible, and easy-to-use in order to facilitate objective data collection and have clinical utility.^19–21^ Scar-assessment methods can be broadly divided into objective and subjective. Quantitative assessments are done with devices created to measure the physical characteristics of the scars. These can be time-consuming and expensive and have not been shown to be better than subjective ones. Subjective tools, on the other hand, provide a qualitative measurement of the scar by the patient and/or the physician. In the absence of one universally recognized “golden standard” for the clinical evaluation of scars, we decided to rely on the 3 systems that have been documented extensively in the literature.

The POSAS^7,8^ is a comprehensive scale that is designed for the evaluation of all types of scars. It evaluates a scar on a scale ranging from 1 to 10 (best to worst) by an observer (on 5 criteria with possible scores range, 5-50) as well as patients' point of view (on 6 criteria with a possible scores range, 6-60).^22–24^ The addition of the patient scale gives the POSAS an important extra dimension for holistic scar evaluation.^7,8,25,26^ It has been found to be more consistent, reliable, and feasible compared with other scales.^20^ In this series, the observer and patient scores for the right breast were 6.91 and 6.82, while those for the left breast were 6.56 and 6.51, respectively.

The VSS is one of the most recognized scar-assessment methods.^27^ It was ﬁrst described by Sullivan in 1990 as a method for burn scar-assessment.^28,29^ In 2000, Nedelec et al^27^ modified it to increase its reliability. VSS takes into consideration vascularity, pigmentation, pliability, and height of the scar and assigns it a score ranging from 0 (best) to 13 (worst). In our series, the mean VSS has been 0.25 for the right and 0.10 for the left breast suggesting a good result 1 year after surgery.

The MSS was created by Beausang et al^30^ and includes an overall visual analog score as well as individual attribute scores.^31^ The scores from the 2 subscales are added together to give an overall score for the scar between 5 (best) to 18 (worst). The MSS uses the term “color mismatch” relative to the surrounding tissue to encompass vascularity and pigmentation, achieving better inter-rater agreement when compared with the VSS.^28^ It is thus able to evaluate a wide range of scars including postoperative scars^32,33^ and has been shown to correlate well with histological and clinical ﬁndings.^30^ In our series, the mean MSS from the right breast was 5.42 and for the left breast was 5.23 which again confirms a good outcome.

### Breast-Q

The BREAST-Q,^9,10^ is a set of validated PROMs (patient-reported outcome measures) used to quantify the impact of aesthetic and reconstructive breast surgery pre- and postoperatively. There are several subscales, eg, those based on health-related quality of life and patient satisfaction scores,^34^ which can be used independently of each other. The range of possible scores is from 0 (worst) to 100 (best). We used the overall satisfaction postoperative subscale for our patients. Our results showed a high satisfaction rate (mean 83.9) which can be interpreted as very good satisfaction of the short scar technique on the overall appearance of the breast.

The single-surgeon design of the study may be thought to introduce a bias. However, these results are from unselected consecutive patients which should minimize any selection bias. Moreover, the strong congruence of patient and clinician scoring of the scars, as well as reassuring BREAST-Q scores goes against the presence of any significant selection bias. There is an argument to include a control group and/or stratify data based on Fitzpatrick skin type and history of previous scar/keloid but that would require a much larger population to have an adequately powered study to gain meaningful results.

In our review, there was good satisfaction with the outcomes from the patient and the physician's perspective. Also, the scar measurement systems demonstrated good correlation among each other and with patients' opinion. These observations let us infer that the Short Scar Technique offers value to the patients, both in terms of satisfactory overall outcomes after breast augmentation as well as satisfactory scars. In other words, our studies showed that it is possible to safely perform breast augmentation through a smaller incision without affecting the final result or the quality of the scar. With this information a surgeon is able to offer breast augmentation to their patients through a smaller incision while being confident that it still provides an appropriate scar and a good overall outcome.

As a matter of fact, this can indeed be considered as advantageous and compelling in the eyes of the many women who are seeking for information about breast augmentation. It is becoming increasingly apparent that, in the age of social media, more and more individuals search online as their first source of information.^35^ The role of discussion forums, individual blogs, and various social media platforms has been steadily increasing in plastic surgery patients (from 46.0% in 2014 to 75.1% in 2019)^35^ as an increasing number of patients look specifically for photographic results of breast augmentation before their scheduled consultation.^35^ We, therefore, feel that it is important, now more than ever, that a surgeon is able to show good results to both his peers as well as prospective patients. While the former occurs in outcomes analyses, reviews, and scientific publications the latter is in social media content. Being able to show beautiful results after breast augmentation with short and good scar quality alongside favorable complications profile, may therefore offer a competitive advantage over peers as well as a marketing opportunity for prospective patients.

## CONCLUSIONS

An inframammary scar for breast augmentation does not have to be as long as classically described, in order to achieve satisfactory outcomes. In our previous work, we showed that it was possible to obtain an equivalent rate of overall complications with a shorter inframammary scar. This review confirms that a shorter IMF scar has a favorable long-term appearance too (as measured by the scar-assessment scales) while providing an overall satisfactory result of the procedure (as measured by the overall postoperative satisfaction subscale of BREAST-Q). For these reasons, we believe that this short scar technique provides added value over the “traditional” inframammary scar, especially for those patients who are conscious about the size and quality of their postoperative scars. In the Social Media era, this may even constitute a marketing tool for the subset of females who look for before and after pictures before choosing their plastic surgeon.
